# Targeting COVID-19 prevention in hemodialysis facilities is associated with a drastic reduction in central venous catheter-related infections

**DOI:** 10.1007/s40620-020-00900-3

**Published:** 2020-12-28

**Authors:** Marco Heidempergher, Gianmarco Sabiu, Maria Antonietta Orani, Giovanni Tripepi, Maurizio Gallieni

**Affiliations:** 1 Nephrology Unit, ASST Fatebenefratelli Sacco, Milano, Italy; 2 School of Nephrology, Università di Milano, Milano, Italy; 3 Clinical Epidemiology and Physiopathology of Renal Diseases and Hypertension, National Research Council (CNR), Institute of Clinical Physiology (IFC), Reggio Calabria, Italy; 4 Department of Biomedical and Clinical Sciences “Luigi Sacco”, Università di Milano, via G.B. Grassi, 74, 20157 Milano, Italy

**Keywords:** Hemodialysis, COVID-19, Central venous catheter, Infection, Vascular access

## Abstract

**Background:**

In hemodialysis (HD) patients, central venous catheter (CVC) related bloodstream infections are a major cause of morbidity and mortality. Hygienic precautions are a key aspect of dialysis care for infection prevention, but they are not sufficient to completely avoid the occurrence of CVC related infections. During the COVID-19 pandemic, hygienic precautions for preventing viral transmission have been markedly reinforced. We evaluated their effects on CVC-related infection rates.

**Methods:**

An observational retrospective study was conducted in two hemodialysis units of the same institution treating 215 chronic hemodialysis patients, 71 of whom are currently (33%) using a CVC. In the CVC cohort, we compared data on catheter-related infection rates during the maximum spread of the COVID-19 pandemic in Italy (February to May 2020) with data from the same period of the previous year and with the whole of 2019.

**Results:**

In 2019, we recorded a catheter-related bloodstream infection (CRBSI) rate of 1.19 (95% CI 0.81–1.68)/1000 days [2.07 (95% CI 1.12–3.52)/1000 days in the Feb-May 2019 period] and a tunnel and exit-site infection rate of 0.82 (95% CI 0.51–1.24)/1000 days [1.04 (95% CI 0.41–2.15)/1000 days in the Feb–May 2019 period]. Infection rates drastically decreased during the COVID-19 pandemic, with just one catheter-related bloodstream infection being recorded. Catheter-related bloodstream infection rates showed a significant reduction to 0.20 (95% CI 0.01–0.9)/1000 days (p < 0.05 and p < 0.005 compared to 2019 and to Feb-May 2019, respectively) and a non-significant reduction in tunnel and exit-site infections to 0.6 (95% CI 0.15–1.6)/1000 days.

**Conclusions:**

The observed 91% reduction in catheter-related bloodstream infections compared to the same period in 2019 [IRR 0.09 (95% CI 0.002–0.64)] and the 83% reduction compared to the whole of 2019 [IRR 0.17 (95% CI 0.004–1.009)] suggest that a stricter implementation of hygienic precautions in the dialysis setting can markedly improve the problem of CVC-related infections.

**Graphic abstract:**

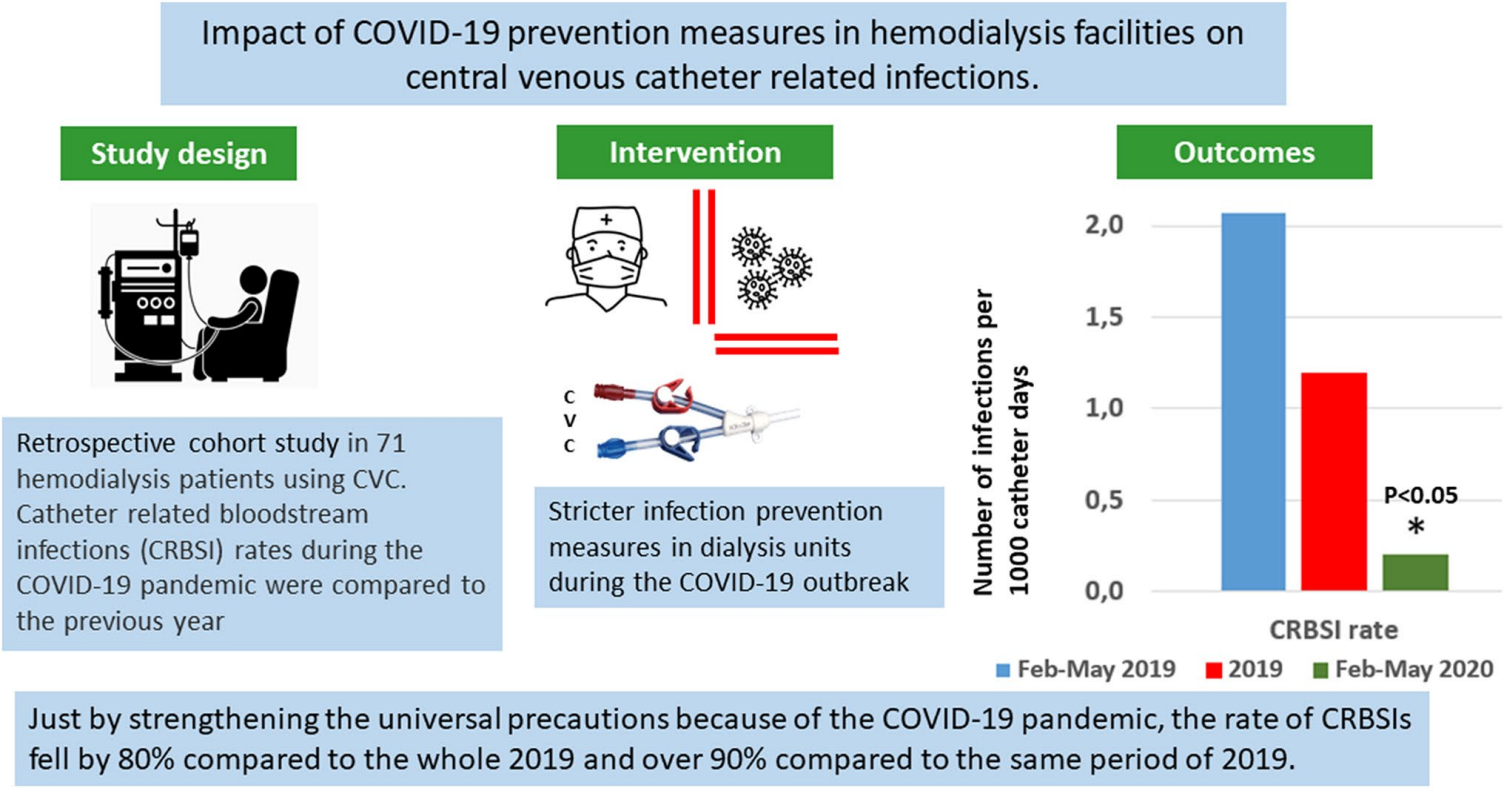

## Introduction

Infections are common complications among patients on chronic hemodialysis (HD), representing the main cause of morbidity and the second cause of death, following cardiovascular events, in this population. Vascular-access-related infections account for nearly 30% of hospitalizations in HD patients. Of all access-related bloodstream infections, 70% occur in patients with central venous catheters (CVC) [1]. In one study, infection-related hospitalization risk was highest for patients dialyzing with a catheter at initiation of dialysis (RR 1.47) and throughout follow-up (RR 2.31), compared to patients with arteriovenous (AV) fistula [2].

This retrospective observational study aims to assess the effect of stricter infection prevention policies adopted during the COVID-19 pandemic on CVC infection rates.

## Methods

When the COVID-19 pandemic began in Italy on February 21st, with Lombardy as the epicenter, we had to reconsider our protocols to ensure safety within our centers and avoid contagion among our patients during hemodialysis. We took many COVID-19 preventive measures [3], but we did not change the management of vascular access for hemodialysis in any way.

### Characteristics of the ASST Fatebenefratelli Sacco dialysis service, affiliated with the University of Milan, Italy

Our academic nephrology and dialysis unit operates in two public hospitals in Milan, one in the city center (Fatebenefratelli) and the other on the outskirts of the city (Sacco). The latter is a teaching hospital and it is, in particular, a national reference center for epidemiological emergencies (SARS, Ebola, and bioterrorism), as well as for the diagnosis and treatment of infectious diseases.

Overall, we treat 215 chronic hemodialysis patients. The prevalence of HD-CVC among our hemodialysis population is nearly 30%. In our hospitals, we have a dedicated vascular access team for each dialysis unit, which is responsible for surveillance programs and treatment of CVC-related infections. The vascular access team includes an expert physician and nurses trained in vascular access care. Since 2018, our vascular access teams have been updating a database with vascular access surveillance data. We take note of all CVC-related infections, the pathogens responsible for them and the antibiotics administered to treat these infections.

### Management and prevention of CVC-related infections in our hospitals

Risk factors for CVC-related infections include previous infections, poor patient hygiene, longer duration of catheter use, inadequate dialysis, hypoalbuminemia, *S. aureus* nasal carriage, diabetes mellitus, immunocompromised status and hypertension [4]. In addition, all catheter manipulations, such as the connection and disconnection phases before and after hemodialysis, are at risk for the migration of pathogens into the bloodstream. Since we are only partially able to intervene on the individual risk factors of patients, it is crucial to minimize the risk during catheter handling. In our hemodialysis units we follow the European Renal Best Practice (ERBP) 2010 guidelines for the prevention of catheter-related infections [5].

Hygienic precautions, using sterile material, are normally applied by caregivers whenever a HD-CVC is manipulated, connected or disconnected to the artificial kidney. Whenever nurses handle the catheter, they and the patient wear a surgical mask; nurses always wear sterile gloves during the manipulation; the skin around the exit-site of the catheter and the catheter itself are always disinfected with chlorhexidine 2% before the use of the catheter [5].

We compared data on CVC-related infections during the period of maximum spread of the COVID-19 pandemic in Italy with data from the same period in 2019.

Extensive spread of the COVID-19 infection was recognized in Italy on Friday 21st, February. We, therefore, set our observation period from the following Monday (24th February) until Friday 15th May because when the “lockdown” in Italy ended on 18th May the number of hospitalizations and active cases of COVID-19 were markedly reduced. Considering the short observation period and, therefore, the risk of over- or underestimating the infection rate compared to 2019, we also compared the COVID-era infection rates with those for the whole of 2019.

We measured infection rates, according to the recent vascular access KDOQI guidelines, as the number of infections per 1000 days of catheter permanence [6]. As a measure of the increased level of universal precautions, we calculated the amount of hydroalcoholic solution that had been ordered from the hospital pharmacy during the observed periods. In 2019, handwashing was the reference clinical practice infection prevention behavior in the dialysis unit.

### Statistical analysis

Data were expressed as means ± standard deviation when normally distributed or as median [interquartile range], or both. The parametric Student’s t-test was used to compare normally distributed datasets. For the calculation of the confidence intervals of the various infection rates examined we used the Mid-p exact test. We then evaluated the statistical significance of the difference between the various rates with the two-sided mid-p-value. The chi-square test was used to compare the catheter permanence in the two dialysis units.

The incidence rate ratio (IRR) was calculated as the ratio of two incidence rates, which in turn are defined as the number of events divided by the person-time at risk.

### Ethical standards, patient consent and ethics committee approval

The study was conducted in accordance with the ethical standards of the committee responsible for human experimentation (institutional and national) and with the Helsinki Declaration of 1964 and its after amendments. The study was approved by the local ethics committee and informed consent was obtained from patients for the anonymous use of their clinical data.

## Results

### Patients and CVC baseline characteristics during the COVID-19 period

At the time of the COVID-19 outbreak 215 hemodialysis patients were being treated in our units. One third (71/215) of all prevalent hemodialysis patients used catheters. Cuffed tunneled central venous catheters accounted for 80% of all catheters (57/71). The internal jugular vein was the site of CVC placement in 94% of the total (67/71). More details are reported in Table 1.

**Table 1:** Characteristics of patients and their central venous catheters (CVCs), February 2020

	Fatebenefratelli Hospital	Luigi Sacco Hospital	Total
Patients with CVC	31	40	71
Age mean ± SD (Years)	68.7 ± 14.4	67.7 ± 17.9	68.1 ± 16.5
Sex (M/F)	16/15	27/13	43/28
CVC parameters			
Tunneled CVC—n. (%)	27 (87.1%)	30 (75.0%)	57 (80.3%)
Non tunneled CVC—n. (%)	4 (12.9%)	10 (25.0%)	14 (19.7%)
IJV CVC—n. (%)	30 (96.8%)	37 (92.5%)	67 (94.4%)
Femoral CVC—n. (%)	1 (3.2%)	3 (7.5%)	4 (5.6%)
CVC vintage (days) mean ± SD - Median [IQR]	687 ± 738 - 505 [159–800]	460 ± 574 - 279 [102–659]	562 ± 655 - 315 [128–674]
CVC vintage > 1 Year—n. (%)	16 (52%)	18 (45%)	34 (48%)
CVC vintage > 2 Years—n. (%)	10 (32%)	7 (18%)	17 (24%)

*IJV* internal jugular vein

### 24th February-15th May, 2019 infection rates

Data regarding the rate of CVC-related infections in the same 2019 time interval in which the 2020 COVID-19 pandemic reached its maximum spread in Italy are reported in Table 2. The period under consideration runs from 24th February to 15th May, 2019 (81 days, since 2020 is a leap year). Table 3 reports data for the whole of 2019.

**Table 2:** February-May 2019 catheter-related infection rates, reported as number of infections/1000 days of utilization

	“Fatebenefratelli” Unit—(95% CI)	“Luigi Sacco” Unit—(95% CI)	Total—(95% CI)
HD-CVC	44	53	97
T-CVC/NT-CVC	37/7	39/14	76/21
IJV CVC/Femoral CVC	40/4	44/9	84/13
Total HD-CVC			
CVC permanence (days)	2549	3247	5796
Exit-site and tunnel infection rate	0.39 (0.01–1.93)	1.54 (0.56–3.41)	1.04 (0.41–2.15)
CRBSI rate	2.35 (0.95–4.89)	1.85 (0.74–3.84)	2.07 (1.12–3.52)
Tunneled CVC			
T-CVC permanence (days)	2248	2706	4954
Exit-site & tunnel infection rate	0.4 (0.02–1.19)	1.85 (0.67–4.09)	1.21 (0.49–2.51)
CRBSI rate	2.22 (0.81–4.93)	2.21 (0.89–4.61)	2.22 (1.16–3.85)
NT-CVC			
Cumulative CVC permanence (days)	301	574	875
Exit-site and tunnel infection rate	0	0	0
CRBSI rate	3.32 (0.16–16.3)	0	1.14 (0.05–5.63)

*CVC* central venous catheter, *HD* hemodialysis, *IJV* internal jugular vein, *T* tunneled, *NT* Non-tunneled, *CRBSI* catheter-related bloodstream infection

**Table 3:** Dialysis catheter-related infection rates in 2019, reported as number of infections/1000 days of utilization

	“Fatebenefratelli” Unit—(95% CI)	“Luigi Sacco” Unit—(95% CI)	Total—(95% CI)
HD-CVC	75	97	172
T-CVC/NT-CVC	53/22	41/56	94/78
IJV / Femoral CVC	65/10	67/30	132/40
Total HD-CVC			
CVC permanence (days)	10,589	13,826	24,415
Exit-site and tunnel infection rate	0.19 (0.03–0.62)	1.30 (0.79–2.01)	0.82 (0.51–1.24)
CRBSI rate	1.51 (0.89–2.42)	0.94 (0.52–1.56)	1.19 (0.81–1.68)
Tunneled CVC			
T-CVC utilization (days)	10,015	11,835	21,850
Exit-site and tunnel infection rate	0.20 (0.03–0.65)	1.52 (0.93–2.35)	0.92 (0.57–1.38)
CRBSI rate	1.30 (0.72–2.16)	0.76 (0.37–1.39)	1.01 (0.64–1.49)
NT-CVC			
Cumulative CVC permanence (days)	574	1991	2565
Exit-site and tunnel infection rate	0	0	0
CRBSI rate	5.23 (1.3–14.2)	2.01 (0.63–4.84)	2.73 (1.19–5.39)

*CVC* central venous catheter, *HD* hemodialysis, *IJV* internal jugular vein, *T* tunneled, *NT* Non-tunneled, *CRBSI* catheter-related bloodstream infection

In the selected February-May 2019 period, catheter-related bloodstream infection (CRBSI) rates are, in both dialysis units, higher than in the rest of 2019. On the other hand, data on the rate of exit-site and tunnel infections are fairly consistent. The catheter-related bloodstream infection rate for non-tunneled central venous catheters (NT-CVCs) was higher among the patients at the “Fatebenefratelli Hospital”. When comparing the baseline data of the two centers, the most important difference involves the use of non-tunneled central venous catheters, which is significantly higher at the “Ospedale Sacco” during the observation period (p < 0.00001), similar to what was observed throughout 2019.

### 2019 Infection rates

In 2019, we recorded a cumulative catheter-related bloodstream infection rate of 1.19/1000 days and a cumulative tunnel and exit-site infection rate of 0.82/1000 days (Table 3). In 2019 there was a single relevant difference in the clinical management of CVCs between the two hemodialysis units: the choice of the CVC lock solution. At the “Sacco” Hospital, 46.7% citrate was the first choice, while, on the contrary, the “Fatebenefratelli” Hospital used heparin. Since January 2020, both dialysis units have been using 46.7% citrate. Overall, in 2019 CVC-related infection rates were 50% higher among the Fatebenefratelli hospital patients. This difference may be related to the different catheter lock solution policy, although it was not statistically significant with the available sample size (1.51/1000 days vs 0.94/1000 days; p = NS). We recorded a stronger difference, again a non-significant one, in the incidence of catheter-related bloodstream infections in patients with temporary catheters between the two hospitals: it was over twofold higher in Fatebenefratelli hospital patients than in Sacco hospital patients (5.23/1000 days vs 2.01/1000 days; p = NS).

**Fig. 1:**
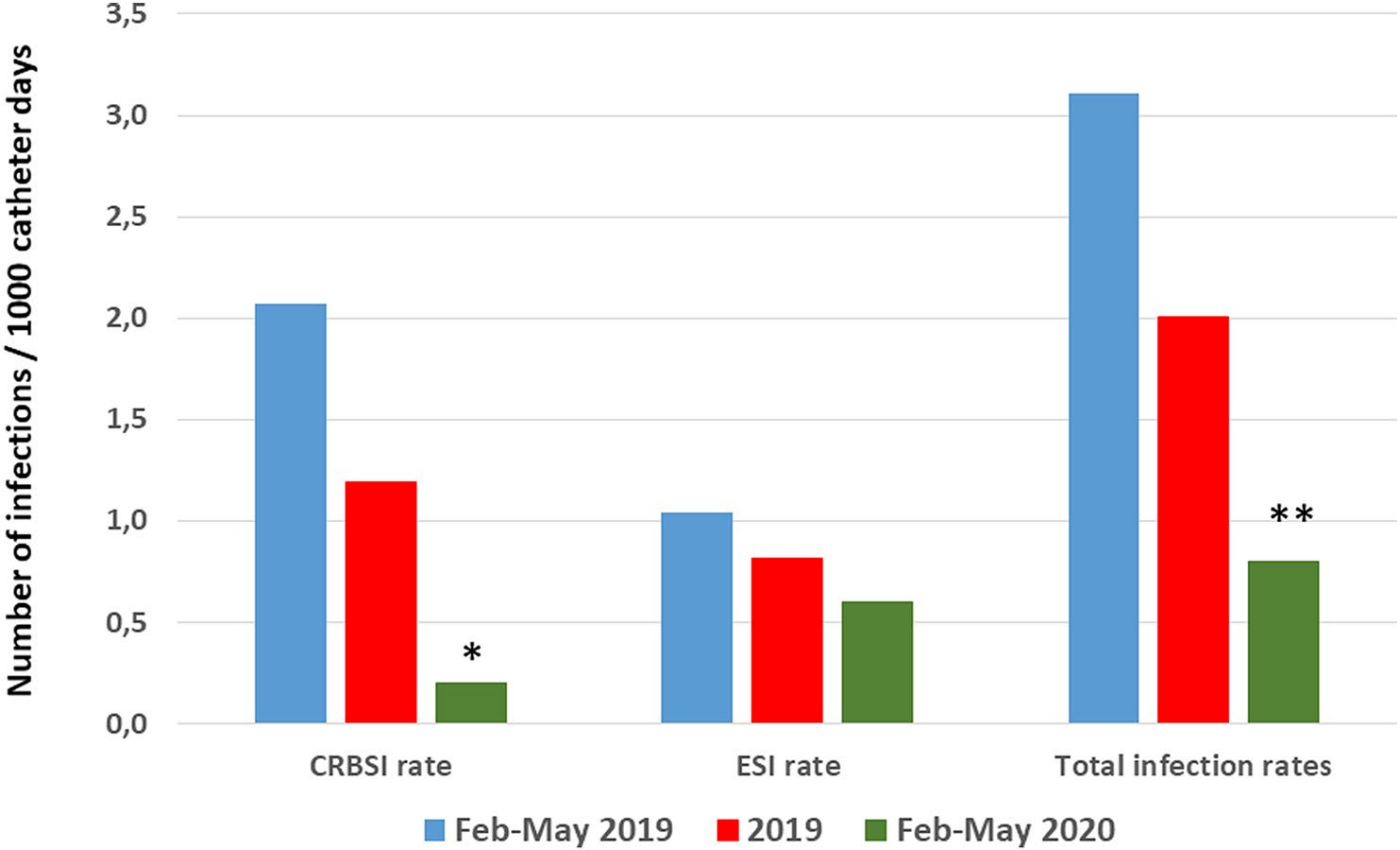
CVC-related infection rates expressed as number of infections/1000 catheter days in the three periods considered: Feb-May 2019, whole of 2019, Feb-May 2020. *CRBSI* Catheter-related blood-stream infection, *ESI* exit site infection

Figure 1 summarizes the comparison of the CVC-related infection rates in the three periods we considered. More detailed information, including 95% confidence intervals of the infection rates, are provided in Table 3.

### COVID-19 period infection rates

During the 81 days from the beginning of the COVID-19 outbreak in Lombardy until mid-May 2020, four patients died for reasons other than catheter-related bloodstream infections and seven catheters were removed.

Infection rates, in particular catheter-related bloodstream infections, drastically decreased (Table 4). Indeed, we only recorded one catheter-related bloodstream infection among the 71 patients considered, which affected a patient who was already waiting to replace his tunneled CVC because of a suspected methicillin-resistant S. Aureus colonization.

**Table 4:** COVID-19 period catheter-related infection rates, reported as number of infections/1000 days of utilization

	“Fatebenefratelli” Unit—(95% CI)	“Luigi Sacco” Unit—(95% CI)	Total—(95% CI)
Total HD-CVC			
N. of CVC	31	40	71
CVC permanence (days)	2286	2744	5031
Exit-site and tunnel infection rate	0.44 (0.2–2.15)	0.73 (0.12–2.40)	0.60 (0.15–1.62)
CRBSI rate	0.44 (0.02–2.15)	0	0.20 (0.01–0.9)
Tunneled CVC			
N. of CVC	27	30	57
TC CVC permanence (days)	2137	2269	4407
Exit-site and tunnel infection rate	0.47 (0.02–2.30)	0.88 (0.14–2.91)	0.68 (0.17–1.85)
CRBSI rate	0.47 (0.02–2.30)	0	0.23 (0.01–0.9)
Non-tunneled CVC			
N. of CVC	4	10	14
NT-CVC permanence (days)	149	475	624
Exit-site and tunnel infection rate	0	0	0
CRBSI rate	0	0	0

*CVC* central venous catheter, *HD* hemodialysis, *IJV* internal jugular vein, *T* tunneled, *NT* Non-tunneled, *CRBSI* catheter-related bloodstream infection

### Infection rates in comparison

Compared to the same period of the previous year, we observed a lower incidence in all infection rates. The cumulative catheter-related bloodstream infection rate, the cumulative exit-site infection rate and the single unit infection rates all decreased, as shown in Table 5. In particular, the cumulative catheter-related bloodstream infection rate shows a drop from 2.07/1000 days to 0.20/1000 days (p = 0.004) [IRR 0.09 (95% CI 0.002–0.64)].

**Table 5:** COVID-19 period vs. Feb-May 2019 (top panel) and entire 2019 (bottom panel) catheter-related infection rates (number of infections/1000 catheter days) at the two dialysis units of ASST Fatebenefratelli Sacco

	Feb–May 2020 COVID-19 era—(CI 95%)	Feb–May 2019—(CI 95%)	Mid-p value
Cumulative			
CRBSI rate	0.20 (0.01–0.9)	2.07 (1.12–3.52)	p = 0.004
Exit-site infection rate	0.60 (0.15–1.62)	1.04 (0.41–2.15)	p = 0.46
Total infections	0.80 (0.25–1.91)	3.11 (1.89–4.81)	p = 0.007
Sacco Hospital			
CRBSI rate	0	1.85 (0.74–3.84)	p = 0.02
Exit-site infection rate	0.73 (0.12–2.40)	1.54 (0.56–3.41)	p = 0.39
Total infections	0.73 (0.12–2.40)	3.39 (1.78–5.88)	p = 0.027
Fatebenefratelli Hospital			
CRBSI rate	0.44 (0.02–2.15)	2.35 (0.95–4.89)	p = 0.09
Exit-site infection rate	0.44 (0.02–2.15)	0.39 (0.01–1.93)	p = 0.94
Total infections	0.87 (0.14–2.89)	2.75 (1.20–5.43)	p = 0.14

	Feb-May 2020 COVID-19 era (95% CI)	2019 (95% CI)	Mid-p value
Cumulative			
CRBSI rate	0.20 (0.009–0.09)	1.19 (0.81–1.68)	p = 0.029
Exit-site infection rate	0.60 (0.15–1.62)	0.82 (0.51–1.24)	p = 0.65
Total infections	0.80 (0.25–1.91)	2.01 (1.50–2.63)	p = 0.05
Sacco Hospital			
CRBSI rate	0	1.30 (0.79–2.01)	p = 0.09
Exit-site infection rate	0.73 (0.12–2.40)	0.94 (0.52–1.56)	p = 0.46
Total infections	0.73 (0.12–2.40)	2.24 (1.55–3.14)	p = 0.09
Fatebenefratelli Hospital			
CRBSI rate	0.44 (0.02–2.15)	1.51 (0.89–2.42)	p = 0.2
Exit-site infection rate	0.44 (0.02–2.15)	0.19 (0.03–0.62)	p = 0.52
Total infections	0.87 (0.14–2.89)	1.70 (1.03–2.63)	p = 0.39

Compared to the whole of 2019, the differences are less striking, but still significant. Indeed, during the period under review, from 24^th^ February to 15^th^ May, 2019, the infection rates were higher than in the rest of the year. The cumulative catheter-related bloodstream infection rate showed a reduction from 1.19/1000 days in2019 to 0.20/1000 days in the COVID-era (p = 0.029) [IRR 0.17 (95% CI 0.004–1.009)]. The cumulative catheter-related bloodstream infection rate of tunneled CVCs fell from 1.01/1000 days to 0.23/1000 days (p = 0.09). Moreover, the “Sacco Hospital” reported no catheter-related bloodstream infections at all (0/1000 days vs 0.94/1000 days; p = 0.09). Although not statistically significant, an interesting finding was the drop in non-tunneled catheter-related bloodstream infections (2.73/1000 days vs 0/1000 days; p = NS), especially at the “Fatebenefratelli Hospital” where the reduction was even higher (5.23/1000 days vs 0/1000 days; p = NS). The cumulative exit-site and tunnel infection rate slightly decreased as well (0.82/1000 days vs 0.60/1000 days). Catheter-related infection rates in 2018 were similar to those in 2019 (exit site 2.65/1000 days; catheter-related bloodstream infections 0.88/1000 days).

There were four deaths during the observation period, none of them due to catheter-related infections, which is similar to the six deaths observed in the same period in 2019 (one related to a catheter-related bloodstream infection).

### Use of hydroalcoholic solution

The adoption of hydroalcoholic hand sanitizer increased markedly from zero to 37 L when comparing the February-May period of 2019 to 2020. In 2019, frequent handwashing was recommended and practiced, while in 2020 the consistent use of hand sanitizer was implemented.

## Discussion

Among HD patients, those with CVC experience a much higher risk of death, infection, cardiovascular events, and hospitalization compared with patients who undergo hemodialysis with an arteriovenous fistula or a graft. A two–threefold higher risk of fatal and nonfatal infections has been reported [7] and in some observational studies, rates of bloodstream infection are even 10 times higher [8].

Catheter-related bloodstream infections, exit-site infections, and tunnel infections are frequent complications related to HD-CVC. Catheter-related bloodstream infection is the most serious complication in patients on hemodialysis with prolonged CVC dependence, and it can lead to a fatal outcome [9]. No specific clinical parameter can predict tunneled hemodialysis CVC-related infection [10].

The rate of hemodialysis catheter-related bloodstream infections reported in literature ranges between 0.8 and 5.5 per 1000 catheter-days [11, 12]. According to DOPPS data based on a limited number of patients, in the United States (US), nearly 70% of patients initiate HD with a CVC, and approximately 15% of all prevalent hemodialysis patients use catheters. In Europe nearly 50% of patients start HD with a CVC, and the prevalence of CVC use varies among countries between 15% (Germany) and 38% (Belgium) [13]. DOPPS data are precious, but they may underestimate the incident and prevalent use of CVCs. Even considering optimistic DOPPS data, the number of patients exposed to the risk of catheter-related bloodstream infections is very high. Prevention of these infections to minimize their incidence is, therefore, a key aspect of dialysis care. In this perspective, KDOQI 2019 vascular access guidelines suggest incorporating an infection surveillance program into clinical care. Guidelines also underline the primary importance of a dedicated team with experience in vascular access care in order to implement effective surveillance programs so as to prevent and manage vascular access issues such as infections [6]. Indeed, having a dedicated vascular access staff for the management and prevention of CVC-related infections reduces CVC failure rates and death from sepsis [14]. It also reduces the number of catheter-dependent patients, with a reported decrease of 80%, and therefore costs [15].

Our study shows consistent reductions in catheter-related bloodstream infections in two dialysis facilities of a single institution, immediately following the COVID-19 outbreak. In the period from 24th February to 15th May, 2020, the rate of catheter-related bloodstream infections in the two dialysis units fell by almost 90%, compared to the same period in 2019. The overall baseline rate in 2019 (1.19 CRBSIs per 1000 catheter days) was not at the desired goal of fewer than 1/1000 catheter days, despite educational efforts and the use of antimicrobial locks. Although the start of hypertonic citrate lock solution in one dialysis unit previously using heparin lock may have contributed to reducing the catheter-related bloodstream infection rate, the improved hygienic precautions account for most of the beneficial effect. Comparing the COVID-era with the whole of 2019, we observed an 80% reduction in the incidence of catheter-related bloodstream infections, with a peak reduction of 100% in catheter-related bloodstream infections of non-tunneled central venous catheters, which are known to have higher infection rates compared to tunneled central venous catheters [1, 11, 16], although a recent report indicated that tunneled central venous catheters and pre-curved non-tunneled central venous catheters showed no difference in reaching the combined endpoint of catheter-related infections and catheter malfunction [17]. There were no differences in terms of exit-site infections, but these are known to be more related to other factors rather than catheter manipulation [18]. Moreover, despite previous findings that catheter-related infections are linked to seasonality, with a higher incidence during the summer [19], we observed a higher incidence of catheter-related bloodstream infections in Feb-May 2019 compared to the whole of 2019. The lack of seasonal changes in our cohort limits this confounding factor in the analysis of 2020.

Limitations of our study are represented by the small sample of patients, due to the single-center design of the study, the short observation time and the before-after design of the study. On the other hand, CVC care and overall patient management is unlikely to have changed in the same dialysis units, giving strength to our results. Since there was no change in the management of hemodialysis catheters, it is useful to understand how and why these results were achieved. The two hospitals considered are located within the metropolitan area of Milan, the capital of Lombardy, the hardest-hit region by the COVID-19 pandemic in Italy. We hypothesize that the health care staff, the nurses and doctors, facing such a dramatic event changed their approach towards the way they work. The level of attention was maximized. Fear of spread of contagion of COVID-19 among patients and healthcare workers maximized the effectiveness of common patient care with regard to the prevention of other infections.

Indeed, hygiene standards have never been so stringent. The use of surgical masks by both the patient and the nurse when manipulating the catheter, a good practice that is routinely observed, may have been followed more accurately during this period due to the fear of spreading droplets. The simple washing of hands before the outpatient enters the dialysis unit is often taken for granted, but in everyday life it is not always done properly. Instead, during the COVID-19 pandemic patients and healthcare staff washed their hands more often and more accurately than they did before. The adoption and use of considerable amounts of hand sanitizer probably contributed to the improved results in infection prevention observed in this study.

Additional countermeasures were taken, such as elimination of the mid-dialysis snack for nurses and patients, even more accurate sanitization of the rooms, and the elimination of blankets. These factors may also have played a role in the reduction of infections [3].

We believe that our findings support the effectiveness of hygienic precautions and usual HD-CVC care, which probably does not need further implementation, but just greater attention. This study lends support to the concept that significant reductions in the current CVC-related infection rates among patients undergoing hemodialysis are achievable. As previously shown in other studies [20–23], prevention of infection by good hygiene, proper handling of catheters, and clean dressing may be the best approach for decreasing the incidence of infectious complications of catheters.

Even when encouraging, observational data are potentially biased and need to be tested in a randomized clinical trial (RCT). However, the design of an RCT with different hygienic precautions standards could be ethically questionable, and (patients would be challenging to include) when proposing different levels of infection prevention. ???

Nevertheless, our results could stimulate further research. When the COVID-19 pandemic is over, a trial could randomly compare the maintenance of the current strict measures of infection prevention versus a less rigorous approach in different shifts or rooms of the same dialysis unit. This study could verify the impact of strict hygienic precautions on infection rates and result in recommendations for their widespread implementation.

In summary, prevention of infections in hemodialysis can be improved. Stricter adherence to recommended prevention practices focused on catheter care and healthcare staff and patient hygiene can further minimize catheter-related infections. COVID-19 may have reminded us of one of the most basic lesson in medicine: hygiene first.

## Data Availability

The datasets supporting the study findings are deposited in the public repository OSF and are available at the following web link: https://osf.io/9zh3c/.
